# Assessment of Reporting Quality of Drug-Related Randomized Controlled Trials Conducted in India and Published in MEDLINE-Indexed Indian Journals Over a Decade: A Systematic Review

**DOI:** 10.7759/cureus.34353

**Published:** 2023-01-29

**Authors:** Madhusudan P Singh, Meenalotchini G Prakash, Nitin R Gaikwad, Yogendra N Keche, Suryaprakash Dhaneria

**Affiliations:** 1 Clinical Pharmacology and Therapeutics, All India Institute of Medical Sciences, Raipur, Raipur, IND; 2 Pharmacology and Therapeutics, All India Institute of Medical Sciences, Raipur, Raipur, IND; 3 Pharmacology and Therapeutics, Ruxmaniben Deepchand Gardi Medical College, Ujjain, IND

**Keywords:** adherence, compliance, consort 2010 statement, randomized controlled trials, reporting quality

## Abstract

Poorly published trials may result in biased and erroneous healthcare decisions. We conducted this systematic review to evaluate the reporting quality of drug-related randomized controlled trials (RCTs) conducted in India and published in MEDLINE-indexed Indian journals over a decade (between January 1, 2011, and December 31, 2020), as per the Consolidated Standards of Reporting Trials (CONSORT) Checklist 2010.

An extensive literature search was conducted using the terms “Randomized controlled trial AND India.” The full-length papers were extracted for RCTs related to drugs. Two independent investigators assessed each article against the checklist containing 37 criteria. Each article was scored 1 or 0 against each criterion which was finally summed up and evaluated.

None of the articles fulfilled all 37 criteria. A compliance rate of >75% was seen in only 15.5% of articles. More than 75% of articles fulfilled a minimum of 16 criteria. Major checklist points observed to be deficient were “important changes to methods after trial commencement” (7%), “interim analysis and stopping guidelines” (7%), and “description of similarity of interventions while blinding” (4%).

There remains ample room for improvement regarding research methodology and manuscript preparation in India. Moreover, journals should stringently implement the CONSORT Checklist 2010 to enhance the standard and quality of publications.

## Introduction and background

A well‑designed, executed, and analyzed randomized controlled trial (RCT) provides a high level of evidence evaluating the effectiveness of healthcare interventions and efficient translation or reliable generation of research data. It provides the fundamentals of evidence‑based medicine and is considered highly reliable data for wider use in the general population. The findings of RCTs assist regulatory bodies in developing or revising national health policy, as well as approving any innovative or experimental treatment. Therefore, a clinical trial must be conducted with a well-designed protocol and reported following all standards. It necessitates a high-quality reporting methodology adhering to the highest possible level, allowing for clarity, as well as enabling readers to evaluate trial results objectively. RCTs can have a significant and immediate impact on patient care. Furthermore, physicians and researchers must consider the quality of methods to make an informed judgment about the validity of the clinical trial [1,2].

The Consolidated Standards of Reporting Trials (CONSORT) Statement was developed in 1996 by a group of researchers, epidemiologists, methodologists, and statisticians to ensure high-quality reporting standards of RCTs in a clear, transparent, and complete manner. This statement was updated twice, first in 2001 and then in 2010, to provide more detailed explanations and elaboration of the CONSORT principles. The CONSORT Statement is a 25-item checklist of 37 criteria that focuses on trial design, analysis, and reporting interpretation [1,2].

As the International Committee of Medical Journal Editors (ICJME) have uniformly accepted the CONSORT guidelines for the publication of RCTs, authors are mandated to submit their work following these guidelines [1]. Reviewers are recommended to ensure that guidelines are being followed for reporting RCTs. Nonetheless, RCTs published in a few Indian medical journals (IMJs) do not meet these criteria.

An observational study by Schultz et al. [3] evaluated whether there is an improvement in editorial policies and the reporting quality of RCTs. Merely one-third of medical journals provided instructions and suggested following the CONSORT guidelines, indicating that RCTs were not carefully monitored to ensure valid and reliable outcomes. Martin et al. [4] found a compelling need to disseminate effective recommendations for methodological implementation and reporting to keep up with the CONSORT design’s extensive use.

Therefore, we performed this systematic review with the primary objective to assess the extent of adherence of RCTs conducted in India and published in IMJs over a decade (between 2011 to 2020) with CONSORT guidelines and identify the areas and domains where reporting could be improved.

## Review

Methodology

This data analysis and systematic review were performed at the Department of Pharmacology, All India Institute of Medical Sciences (AIIMS), Raipur to evaluate the reporting standard as per the CONSORT Checklist 2010. The data collection and analysis were done for six months (from January 1, 2021, to June 30, 2021) after obtaining approval from the Institutional Ethics Committee (1285/IEC-AIIMSRPR/2020).

Search Strategy

**Figure 1:**
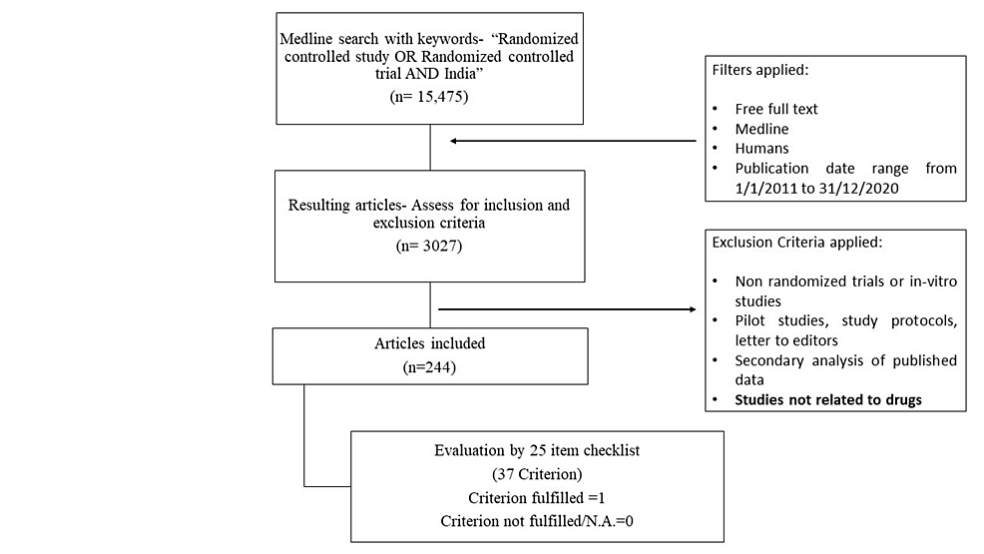
Study flowchart.

The review comprised the following two phases: first, the identification of RCT trials related to drugs, and, second, the data extraction and assessment of RCT quality (Figure 1). A MEDLINE/PubMed search was conducted for all RCTs conducted in India and published in IMJs between January 1, 2011, and December 31, 2020. The keywords “randomized controlled trial” OR “randomized controlled study” AND “India” were used in the search strategy. “Publication date (January 1, 2011, to December 31, 2020),” “Free full text,” and “Medline” as a journal type were the filters used.

Inclusion and Exclusion Criteria

The terms “random,” “randomly,” “randomized,” or “double-blind” were searched for in the titles, abstracts, and keywords of published studies, and those that satisfied these criteria were included in the study. Furthermore, studies in which the participants’ distribution was characterized by the terms “random,” “randomized,” “randomly,” “randomization,” or any other term that indicates that the participants were randomly allocated to different treatment arms were included in the analysis. Articles were recognized as “studies conducted in India” by checking the study site individually in all the studies. Similarly, for recognizing if “studies were published in Indian journals,” the publisher’s address of journals was searched from the master journal list of the Web of science group website (https://mjl.clarivate.com/search-results). Two independent investigators (MPS and MGP) independently selected the articles. Disagreements about study inclusion were resolved through discussion and consensus, and reasons for article exclusion were documented. The analysis excluded non-randomized trials, in-vitro studies, economic analyses, pilot studies, questionnaire reporting, study protocols, non-human studies, diagnostic or screening tests, observational studies, reviews, meta-analyses, secondary analysis of primary data, articles reported only as abstracts or as letters to the editor, studies without methods and with only a summary, and studies not related to drugs.

Data Extraction Strategy

A pilot study was conducted to identify problems and resolve discrepancies in data collection and analysis, which included RCTs published in the year 2019 (i.e., a one-year duration). Two independent investigators (MPS and MGP) conducted the final data extraction for the predefined period articles, similar to the pilot analysis, using the previously generated and pretested data extraction and documentation form according to the CONSORT checklist items.

Scoring

Scoring was done using a 25-item checklist, comprising 37 criteria included in the CONSORT 2010 statement. We analyzed all studies that were included to provide the most scientific evidence. For each criterion’s presence and absence in each article, a score of 1 or 0 was assigned, respectively. The cumulative score for each article was calculated by adding item-by-item scores. Journal and paper characteristics such as journal name, first author name, year of publication, multicenter design, and adequate sample size were also noted.

Data Synthesis and Presentation

Microsoft Excel 2013 and the SPSS package version 25.0 (IBM Corp., Armonk, NY, USA) were used to interpret the data for descriptive statistics. Percentages and 95% confidence intervals (CI) were calculated to analyze the primary outcome. Cohen’s kappa score was calculated for each criterion to determine concurrent readers’ agreement. For each parameter, a kappa score of 0.60 or higher was deemed a significant interobserver agreement. Disagreements were resolved by discussion or consultation with a third reviewer.

Results and discussion

A MEDLINE search for RCTs yielded 15,475 studies, of which 3,027 were identified after the filters were applied. A total of 244 articles were eventually included in our sample after being assessed for inclusion and exclusion criteria. The articles were then assessed for compliance with the CONSORT 2010 Statement using a 25-item checklist.

During data analysis, the interobserver agreement (combined Cohen’s kappa) for all criteria was 0.70, indicating a good agreement between the two observers.

**Figure 2:**
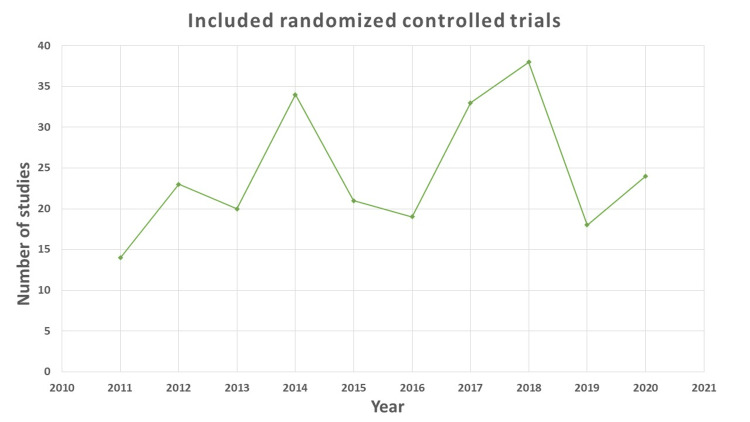
Year-wise frequency of included randomized controlled trials in the study.

None of the articles met all 37 criteria; however, we observed that more than 75% of articles met a minimum of 16 criteria. The majority of the studies included in the analysis were from 2018, supplemented by 2014 and 2017 (Figure 2). Most studies that showed conformity to the CONSORT checklist between 76% and 100% were from 2016 (eight), followed by 2014 (five), 2015 (five), and 2017 (five) (Table 1).

**Table 1:** Year-wise adherence of articles to CONSORT 2010 statement. CONSORT = Consolidated Standards of Reporting Trials

Year	Adherence to CONSORT 2010 Statement			
	0–25% n (%)	26–50% n (%)	51–75% n (%)	76–100% n (%)
2011	0	3 (22%)	9 (64%)	2 (14%)
2012	0	5 (22%)	16 (69%)	2 (9%)
2013	0	2 (10%)	17 (85%)	1 (5%)
2014	0	4 (12%)	25 (74%)	5 (14%)
2015	0	1 (5%)	15 (71%)	5 (24%)
2016	0	3 (16%)	8 (42%)	8 (42%)
2017	0	6 (18%)	22 (67%)	5 (15%)
2018	0	6 (16%)	29 (76%)	3 (8%)
2019	0	3 (16%)	12 (67%)	3 (17%)
2020	0	6 (25%)	17 (71%)	1 (4%)
Total (244)	0	39 (16%)	170 (70%)	35 (14%)

In 14% of the articles, the compliance rate was greater than 75%. We found that the majority of the articles, 70%, were compliant in the 50-75% range, and 16% were compliant in the 50% range (Table 1).

Some of the checklist points were “not applicable” for the studies to comply with, and in which the trials were deficient were “Important changes to methods after trial commencement” (7%), if “Any changes to trial outcomes after the trial commenced” (7%), “Interim analysis & stopping guidelines” (6%), “description of the similarity of interventions while blinding” (4%) and, “Why the trial ended or was stopped” (3%) (Table 2).

**Table 2:** Percentage adherence of RCTs to the CONSORT checklist. RCT = randomized controlled trial; CONSORT = Consolidated Standards of Reporting Trials

Item number	Section/Title	Criteria number	Items	Articles fulfilling the criterion n (%)	95% confidence interval
1	Title and abstract	1	Identification as a randomized trial in the title	144 (59%)	0.59 (0.52-0.65)
		2	Structured summary of trial design, methods, results, and conclusions	233 (95%)	0.95 (0.92-0.97)
Introduction					
2	Background and objectives	3	Scientific background and explanation of the rationale	244 (100%)	1 (0.99-1)
		4	Specific objectives or hypotheses	241 (99%)	0.99 (0.97-1)
Methods					
3	Trial design	5	Description of trial design (such as parallel, factorial) including allocation ratio	210 (86%)	0.86 (0.81-0.90)
		6	Important changes to methods after trial commencement (such as eligibility criteria), with reasons	16 (7%)	0.7 (0.64-0.75)
4	Participants	7	Eligibility criteria for participants	242 (99%)	0.99 (0.97-1)
		8	Settings and locations where the data were collected	205 (84%)	0.84 (0.79-0.88)
5	Interventions	9	The interventions for each group with sufficient details to allow replication, including how and when they were actually administered	238 (98%)	0.98 (0.96-0.99)
6	Outcomes	10	Completely defined pre-specified primary and secondary outcome measures, including how and when they were assessed	227 (93%)	0.93 (0.89-0.96)
		11	Any changes to trial outcomes after the trial commenced, with reasons	12 (5%)	0.5 (0.43-0.56)
7	Sample size	12	How the sample size was determined	120 (49%)	0.49 (0.42-0.55)
		13	When applicable, explanation of any interim analyses and stopping guidelines	15 (6%)	0.6 (0.53-0.66)
8	Randomization: sequence generation	14	The method used to generate the random allocation sequence	176 (72%)	0.72 (0.66-0.77)
		15	Type of randomization; details of any restriction	129 (53%)	0.53 (0.46-0.59)
9	Allocation concealment mechanism	16	The mechanism used to implement the random allocation sequence (such as sequentially numbered containers), describing any steps taken to conceal the sequence until interventions were assigned	106 (43%)	0.43 (0.36-0.49)
10	Implementation	17	Who generated the random allocation sequence, who enrolled participants, and who assigned participants to interventions	95 (39%)	0.39 (0.32-0.45)
11	Blinding	18	If done, who was blinded after assignment to interventions and how	96 (39%)	0.39 (0.32-0.45)
		19	If relevant, description of the similarity of interventions	9 (4%)	0.4 (0.33-0.46)
12	Statistical methods	20	Statistical methods used to compare groups for primary and secondary outcomes	241 (99%)	0.99 (0.97-1)
		21	Methods for additional analyses, such as subgroup analyses and adjusted analyses	47 (19%)	0.19 (0.14-0.23)
Results					
13	Participant flow	22	For each group, the numbers of participants who were randomly assigned, received intended treatment, and were analyzed for the primary outcome	125 (51%)	0.51 (0.44-0.57)
		23	For each group, losses and exclusions after randomization, together with reasons	106 (43%)	0.43 (0.36-0.49)
14	Recruitment	24	Dates defining the periods of recruitment and follow-up	157 (64%)	0.64 (0.57-0.70)
		25	Why the trial ended or was stopped	7 (3%)	0.3 (0.24-0.35)
15	Baseline data	26	A table showing the baseline demographic and clinical characteristics for each group	207 (85%)	0.85 (0.80-0.89)
16	Number allocation	27	For each group, the number of participants (denominator) included in each analysis and whether the analysis was by original assigned groups	224 (92%)	0.92 (0.88-0.95)
17	Outcomes and estimation	28	For each primary and secondary outcome, results for each group, and the estimated effect size and its precision (such as 95% confidence interval)	236 (97%)	0.97 (0.94-0.99)
		29	For binary outcomes, the presentation of both absolute and relative effect sizes is recommended	79 (32%)	0.32 (0.26-0.37)
18	Ancillary analyses	30	Results of any other analyses performed, including subgroup analyses and adjusted analyses, distinguishing pre-specified from exploratory	39 (16%)	0.16 (0.11-0.20)
19	Harms	31	All-important harms or unintended effects in each group	132 (54%)	0.54 (0.47-0.60)
Discussion					
20	Limitations	32	Trial limitations, addressing the sources of potential bias, imprecision, and, if relevant, multiplicity of analyses	181 (74%)	0.74 (0.68-0.79)
21	Generalizability	33	Generalizability (external validity, applicability) of the trial findings	224 (92%)	0.92 (0.88-0.95)
22	Interpretation	34	Interpretation consistent with results, balancing benefits and harms, and considering other relevant evidence	222 (91%)	0.91 (0.87-0.94)
Other information					
23	Registration	35	Registration number and the name of trial registry	77 (32%)	0.32 (0.26-0.37)
24	Protocol	36	Where the full trial protocol can be accessed, if available	62 (25%)	0.25 (0.19-0.30)
25	Funding	37	Sources of funding and other support (such as supply of drugs), the role of funders	235 (95%)	0.95 (0.92-0.97)

We also observed that some of the criteria of the CONSORT statement had a compliance rate of 98-100% in the RCTs included in this study. They were “scientific background and explanation of rationale” (100%), “statistical methods used to compare groups for primary and secondary outcomes” (99%), “specific objectives or hypotheses” (99%), “eligibility criteria for participants” (99%), and the “interventions for each group” (98%) (Table 2).

**Figure 3:**
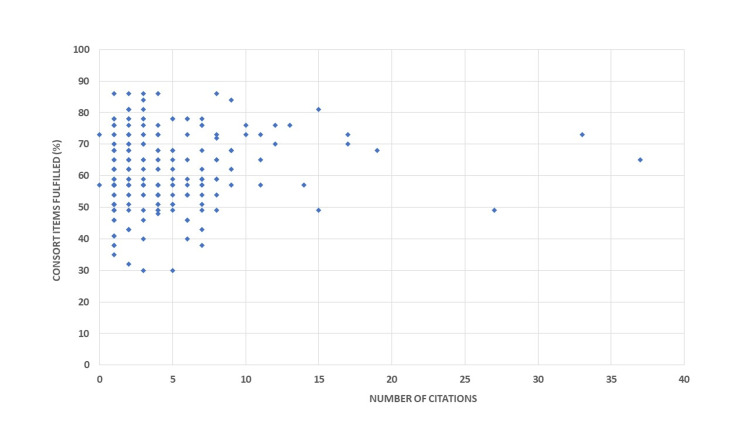
Correlation of adherence of RCTs to the CONSORT checklist and their citation frequency. RCT = randomized controlled trial; CONSORT = Consolidated Standards of Reporting Trials

We also checked for the correlation between the frequency at which these RCTs were cited and their compliance with the CONSORT checklist. We discovered that trials with greater than 50% conformity received more citations, and this association was statistically significant (r = 0.793; p = 0.022) (Figure 3).

Evidence of Effectiveness

RCTs are regarded as the gold standard and their consistent reporting will enhance trial evaluation and interpretation, as well as clarity, retrieval, accurate indexing, and reasonable inclusion in systemic reviews. Clinical experience, thorough planning and coordination of each investigator’s efforts, and a contingency plan for unanticipated problems are essential components of a well-designed and executed RCT. The CONSORT statement was developed to aid in the analysis and critical assessment of RCTs by guiding authors on how to improve their trial reporting. Our systematic review revealed that, despite a 10-year timeframe, none of the drug-related RCTs in India achieved 100% adherence to the CONSORT statement.

In their analysis, Nozomi et al. [5] observed that only 30% of the articles they reviewed mentioned sample size estimation, while 96% of the articles reported statistical methods used to compare groups for primary and secondary outcomes. Nonetheless, in our analysis, 49% and 99% of articles were compliant with these criteria, indicating an increase in adherence to the CONSORT checklist over time. Calculating the appropriate sample size is crucial because it aids in drawing a precise and reliable conclusion to any research [6]. Furthermore, proper statistical analysis and interpretation are needed to effectively communicate research findings, support theories, and provide credibility to the conclusions and results. It is also essential to report accurate p-values and test statistics [7]. It has been observed that even renowned journals such as BMJ and Nature had flawed proportions of 25.0% and 38.0%, respectively, in terms of the presentation of statistical results [8,9].

In their analysis, Hassan et al. [10] discovered that RCTs reported in India had serious methodological errors, such as the lack of power estimates, sample size estimation, and inadequacy to report randomization. In our study, only 49% of articles mentioned sample size estimation, which is one of the criteria in the CONSORT statement. Any study should have at least 80% power (type II error), and power estimation is important as sample size increases when the power of the study is increased from 80% to 90% or 95% [6]. Furthermore, other methodological errors of concern that we observed in our study were types of randomization (53%), methods to implement random allocation sequence (43%), random allocation sequence implementation (39%), and blinding (39%).

In our analysis, we observed that 72% of articles had reported the method used to generate the random allocation sequence, and 53% had reported the type of randomization. Randomization increases the support of evidence for the study by establishing a more evident causal relationship, controlling lurking variables, and reducing any bias in the study.

Wandalkar et al. [11], in their study, discovered a significant disparity between registered protocols with clinical trials registry and the final manuscripts which were published. As a result, item 3b, which states, “Important improvements to methods after trial start” is advantageous. In our study, only 7% of articles reported this statement.

In their research, Shaikh et al. [12] noticed that CONSORT flow diagrams were included in 37.5% of RCTs reported in Indian journals, while in our study, flow diagrams were included in 51% of RCTs. The use of flow diagrams improves the quality of RCT reporting [13]. In addition, it helps to assess the generalizability and validity of the results of RCTs [14]. Hopewell et al. suggested the authors generate CONSORT-specific diagrams and charts using various web-based programs for making it easier to apply [15]. Moreover, there is a need to educate investigators about various guidelines and the development of “writing aid tools” [16].

A meta-analysis conducted by Stevens et al. [17] found insufficient evidence of a relationship between reporting completeness and guideline endorsement. Turner et al. [18] noted significant improvements in just five CONSORT items and composite scores in a Cochrane review, concluding that journals’ endorsement could have a positive impact on an article’s reporting quality.

Goyal et al. [19] analyzed 46 clinical trials published in three Indian journals between 2007 and 2008 and observed that during the interpretation of results, most scientific papers had errors. RCTs are touted as the gold standard for scientific evidence regarding the comparison of drugs or treatment modalities. If these studies are inadequately reported, guidelines that are backed by this evidence may make recommendations based on poorly conducted trials, which may ultimately lead to worsened patient outcomes and erroneous clinical decision-making.

There are several reasons why authors do not follow the CONSORT guidelines. First, “CONSORT endorsing journals” providing specific instructions to follow the CONSORT statement for reporting studies are few in number [15,20,21]. Second, authors often use the wrong checklist extension resulting in discrepancies between the actual manuscript and the checklist [22]. Third, the editorial team, including reviewers, is untrained and ignorant about various reporting guidelines of studies. According to the analysis by Cobo and González, one-third of manuscripts identified as trials by editors and their staff were not actually trials [23]. Hirst and Altman also mentioned that the reviewers did not have adequate guidance about the use of guidelines and checklists [24].

Jauch et al., in their study, found that citation frequency has a high correlation with the ratio of the fulfillment of the CONSORT item checklist (r = 0.6747; p < 0.0001). [25] Similarly, Stevanovic et al. also found a significant relationship between the citation frequency of the trials to the CONSORT statement adherence (r = 0.331; p < 0.001) [26] which is similar to our findings (r = 0.793; p = 0.022). Therefore, we can deduce that overall compliance with CONSORT guidelines is directly proportional to the frequency of citations.

Limitations

Due to the non-availability of institutional access to paid subscription journals at the time of the literature search, only free full-text articles that were published in MEDLINE-indexed journals were included. This resulted in the exclusion of 27% of studies that would have otherwise been included. Further research should be conducted involving a wider variety of publications over a more extended period of time, including paid journals and those indexed in other databases.

## Conclusions

We can conclude from our study that the majority of RCTs were not adherent to the CONSORT 2010 statement. Many published RCTs were found to be inadequate in comprehensive detailing of randomization, random allocation sequence, sampling, and blinding. However, an increase in adherence to the CONSORT checklist with time was seen in reporting sample size estimation and statistical methods.

We have also demonstrated that an increase in adherence to the CONSORT statement is directly proportional to the frequency of citations of the studies. Thus, we suggest authors adhere to the CONSORT statement while publishing RCTs. Moreover, journals and their editorial team should endorse the CONSORT statement and check for articles that comply with the CONSORT statement, as this would further improve the reporting quality of RCTs that would ultimately help make guidelines to take the right clinical decisions for better patient outcomes and to increase the number of citations for the studies.
